# Beneficial Effects of HIV Peptidase Inhibitors on *Fonsecaea pedrosoi*: Promising Compounds to Arrest Key Fungal Biological Processes and Virulence

**DOI:** 10.1371/journal.pone.0003382

**Published:** 2008-10-13

**Authors:** Vanila F. Palmeira, Lucimar F. Kneipp, Sonia Rozental, Celuta S. Alviano, André L. S. Santos

**Affiliations:** 1 Laboratório de Estudos Integrados em Bioquímica Microbiana, Departamento de Microbiologia Geral, Instituto de Microbiologia Prof. Paulo de Góes (IMPPG), Centro de Ciências da Saúde (CCS), Universidade Federal do Rio de Janeiro (UFRJ), Rio de Janeiro, Brazil; 2 Laboratório de Estrutura de Microrganismos, Departamento de Microbiologia Geral, tuto de Microbiologia Prof. Paulo de Góes (IMPPG), Centro de Ciências da Saúde (CCS), Universidade Federal do Rio de Janeiro (UFRJ), Rio de Janeiro, Brazil; 3 Laboratório de Taxonomia, Bioquímica e Bioprospecção de Fungos, Instituto Oswaldo Cruz, Fundação Oswaldo Cruz, Rio de Janeiro, Brazil; 4 Laboratório de Biologia Celular de Fungos, Instituto de Biofísica Carlos Chagas Filho (IBCCF), Centro de Ciências da Saúde (CCS), Universidade Federal do Rio de Janeiro (UFRJ), Rio de Janeiro, Brazil; University of Minnesota, United States of America

## Abstract

**Background:**

*Fonsecaea pedrosoi* is the principal etiologic agent of chromoblastomycosis, a fungal disease whose pathogenic events are poorly understood. Current therapy for chromoblastomycosis is suboptimal due to toxicity of the available therapeutic agents and the emergence of drug resistance. Compounding these problems is the fact that endemic countries and regions are economically poor.

**Purpose and Principal Findings:**

In the present work, we have investigated the effect of human immunodeficiency virus (HIV) peptidase inhibitors (PIs) on the *F. pedrosoi* conidial secreted peptidase, growth, ultrastructure and interaction with different mammalian cells. All the PIs impaired the acidic conidial-derived peptidase activity in a dose-dependent fashion, in which nelfinavir produced the best inhibitory effect. *F. pedrosoi* growth was also significantly reduced upon exposure to PIs, especially nelfinavir and saquinavir. PIs treatment caused profound changes in the conidial ultrastructure as shown by transmission electron microscopy, including invaginations in the cytoplasmic membrane, disorder and detachment of the cell wall, enlargement of fungi cytoplasmic vacuoles, and abnormal cell division. The synergistic action on growth ability between nelfinavir and amphotericin B, when both were used at sub-inhibitory concentrations, was also observed. PIs reduced the adhesion and endocytic indexes during the interaction between conidia and epithelial cells (CHO), fibroblasts or macrophages, in a cell type-dependent manner. Moreover, PIs interfered with the conidia into mycelia transformation when in contact with CHO and with the susceptibility killing by macrophage cells.

**Conclusions/Significance:**

Overall, by providing the first evidence that HIV PIs directly affects *F. pedrosoi* development and virulence, these data add new insights on the wide-spectrum efficacy of HIV PIs, further arguing for the potential chemotherapeutic targets for aspartyl-type peptidase produced by this human pathogen.

## Introduction

Chromoblastomycosis is a chronic, suppurative and progressive mycosis of the skin and subcutaneous tissues [1]. *Fonsecaea pedrosoi*, a polymorphic pathogenic fungus, is considered its most frequent etiologic agent that occurs worldwide but is more commonly found in tropical and subtropical regions of America and Africa, and causes a typical granulomatous inflammatory response, whose degree reflects the immune status of the host. Chromoblastomycosis occurs mainly in farmers and rural workers. It usually affects the upper and the lower limbs, where *F. pedrosoi* gain entrance through the skin by traumatic implantation of conidia and fragments of mycelial cells [1], [2], [3]. Lesions can vary in appearance from flat plaques to warty lesions, and grow slowly into clusters of large hyperkeratotic verrucous plaques with central scarring, ulceration and cystic areas. Limbs can become deformed with secondary lymph edema and keratin necrosis can also result [1]. Other complications of chromoblastomycosis include carcinomatous degeneration, elephantiasis of the affected limbs and secondary bacterial infection [4].

Chromoblastomycosis lesions are recalcitrant and extremely difficult to eradicate. Chemotherapy, surgical excision and/or cryosurgery have been used throughout the years [5], [6], but an effective treatment for chromoblastomycosis has not yet been established. Treatment of the mycosis caused by this agent is unrewarding not only because of the scarcity of effective antifungal agents but also due to the need for prolonged periods of treatment, which in some reports has required extended therapeutic regimens of up to 2 years to obtain a mycological cure [7]. Such treatment is not very effective, producing relapses during therapy and lack of tolerance of antifungal drugs. The disease is usually insidious and the lesions increase slowly but progressively, not responding to the usual treatments and quite often reappearing. Therefore, more targeted use of antifungal agents is therefore required to help minimize future incidences of infection.

In recent years, the introduction of anti-retroviral drugs, particularly aspartyl peptidase inhibitors (PIs) used in the chemotherapy of the human immunodeficiency virus (HIV), into medical practice had led to a marked improvement in the life expectancy of AIDS sufferers by the fall of HIV viremia and by restoring the immune responses with an increase in the number of CD4^+^ T lymphocytes [8], [9], [10], [11]. In addition, the functional upgrading of two critical components of innate antimicrobial immunity, such as neutrophils and monocytes, may contribute to the improved cell-mediated immune responses against opportunistic infections in highly active anti-retroviral therapy (HAART)-treated patients [9], such as candidiasis [12], cryptococcosis [13] and microsporidiosis [14]. Nevertheless, experimental evidences demonstrated that HIV aspartyl-type PIs also exerted a direct inhibitory effect on AIDS-related opportunistic pathogens. In particular, HIV PIs profoundly reduce *Candida albicans* in vitro growth and experimental pathogenicity. The phenomenon is associated with a direct effect of such drugs on the production of *C. albicans* secreted aspartyl peptidases (Saps), which assist the fungus to colonize and invade host tissues, and to evade the host's antimicrobial defense mechanisms [reviewed in 15].

Interestingly, *F. pedrosoi* conidial and mycelial cells extracellularly released aspartyl-type peptidases, when cultured under chemically defined conditions, which were capable of hydrolyzing several proteinaceous substrates including human serum proteins (e.g., albumin, immunoglobulin G and fibrinogen), extracellular matrix components (e.g., laminin, fibronectin and type I collagen) and sialylated glycoproteins (e.g. fetuin and mucin). Taken together, these *F. pedrosoi* secreted peptidases are expected to favor assimilation of nitrogen from different proteinaceous sources and have been proposed as potential virulence attributes, which help the fungal tissue dissemination and/or escape of host immune response [16], [17].

Peptidases participate in several physiological and pathological processes in different cell types. In pathogenic fungi, this class of hydrolytic enzymes directly acts in different steps of the microorganism-host interplay, being considered as virulence factor [15], [18]. Considering all these facts together, we have conducted a study to investigate the direct effect of four different HIV PIs (indinavir, saquinavir, ritonavir and nelfinavir), commonly used in HAART, on the *F. pedrosoi* conidial secreted aspartyl peptidase, growth ability, ultrastructure and interaction of this human pathogen with distinct animal cell lineages in vitro. The possible synergistic effect between PI and antifungal compounds was also available.

## Methods

### Chemicals

Saquinavir and nelfinavir were obtained from Hoffmann-La Roche AG (Grenzach-Wyhlen, Germany), indinavir was from Merck Sharp & Dohme GmbH (Haar, Germany) and ritonavir from Abbot Park (Illinois, USA), which were dissolved in absolute methanol to obtain a final concentration of 20 mM and stored at −20°C until use. Amphotericin B, itraconazole, bovine serum albumin (BSA), propidium iodide, dimethylsulfoxide (DMSO), 3-(4,5-dimethylthiazol-2-yl)-2,5-diphenyl tetrazolium bromide dye (MTT), *trans*-epoxysuccinyl l-leucylamido-(4-guanidino) butane (E-64), phenylmethylsulphonyl fluoride (PMSF), pepstatin A and 1,10-phenanthroline were purchased from Sigma Chemical Co. (St Louis, USA). Media constituents, reagents used in electrophoresis and buffer components were purchased from Amersham Life Science (Little Chalfont, UK). All other reagents were analytical grade.

### Microorganisms and growth condition

A pathogenic strain of *Fonsecaea pedrosoi* (5VLP), isolated from a human patient with chromoblastomycosis [19], was used in all parts of the present work. This *F. pedrosoi* strain is being investigated by our research group in order to understand its biological, biochemical and infectious properties [reviewed in 3]. Stock cultures were maintained on Sabouraud dextrose agar under mineral oil and kept at 4°C. Transfers were made at 6-month intervals. For conidium formation [20], cultures were incubated for 5 days under constant agitation (200 rpm) at room temperature in 250-ml Erlenmeyer flask containing 100 ml of Czapek-Dox chemically defined medium containing (g/l): sucrose, 30; NaNO_3_, 2; K_2_HPO_4_, 1; MgSO_4_.7H_2_O, 0.5; KCl, 0.5; FeSO_4_.7H_2_O, 0.01; pH 5.5. Additionally, two other clinical isolates (designated as 11428 and Magé strains) were used to demonstrate that the secretion of peptidase activity is a common feature of *F. pedrosoi* species, instead of being strain-specific.

### Evaluation of conidia growth and viability

Cellular growth was estimated by counting the conidial cells in a Neubauer chamber. The viability of conidia was assessed by colony-forming unit (CFU) measurements or propidium iodide staining. In this last methodology, cell death due to a loss in cell membrane integrity of drug-treatment *F. pedrosoi* was assessed by flow cytometry measuring the level of propidium iodide uptake into damage conidia.

### Cell-free culture supernatant and protein content

The cultures were centrifuged (4000 *g*, 10 minutes, 4°C) and the supernatants were filtered in a 0.22-µm membrane (Millipore). The cell-free culture supernatants were concentrated 100-fold in a 10,000 molecular weight cut-off Amicon micropartition system (Beverly, MA, USA) [16]. Protein concentration was determined by the method described by Lowry et al. [21], using BSA as standard. Samples containing 5 µg of released proteins were added to 10 µl of sodium dodecyl sulfate-polyacrylamide gels electrophoresis (SDS–PAGE) sample buffer (125 mM Tris, pH 6.8, 4% SDS, 20% glycerol, 0.002% bromophenol blue) supplemented with 5% β-mercaptoethanol, followed by heating at 100°C, for 5 minutes. Proteins were analyzed in 12% SDS–PAGE using the method described by Laemmli [22]. Electrophoresis was carried out at 4°C, at 120 V, and the gels were silver stained. The molecular masses of sample polypeptides were calculated from mobility of GIBCO BRL (Grand Island, NY, USA) molecular mass standards.

### Quantitative proteolytic activity assay

The extracellular proteolytic activity was measured spectrophotometrically using the substrate BSA, according to the method described by Buroker-Kilgore and Wang [23]. Briefly, 20 µl of concentrated supernatant (5 µg), BSA (0.5 mg/ml) and 10 mM sodium citrate (pH 2.0–5.0) was added to a microcentrifuge tube (350 µl) and incubated for 1 hour at 37°C. After incubation, three aliquots (100 µl each) of the reaction mixture were transferred to wells on a microtiter plate containing 50 µl of water and 100 µl of a Coomassie solution (0.025% Coomassie brilliant blue G-250, 11.75% ethanol and 21.25% phosphoric acid). A control, where the substrate was added just after the reactions were stopped, was used as blank. After 10 min to allow dye binding, the plate was read on a Molecular Devices Thermomax microplate reader at an absorbance of 595 nm. One unit of enzyme activity was defined as the amount of enzyme that caused an increase of 0.001 in absorbance unit, under standard assay conditions [16], [17]. Alternatively, the concentrated supernatant was pre-incubated for 20 minutes at 37°C in the presence or absence of the following PIs: PMSF (1 mM), E-64 (1 µM), 1,10-phenanthroline (1 mM), pepstatin A (1, 5, 10 and 20 µM), indinavir, saquinavir, ritonavir and nelfinavir (50, 100 and 200 µM). In this last case, the results were expressed as relative percentage of activity with PIs subtracted from the activity without inhibitors. Besides, the reaction mixtures were applied on SDS-PAGE to demonstrate the fragments correspondent to the BSA hydrolysis; in this situation, the gels were stained with Coomassie blue R-250 solution [17].

### Effect of aspartyl PIs on the fungal development

The culture of *F. pedrosoi* conidia was centrifuged at 4000 *g* for 10 minutes at 4°C. The cells were washed three times in phosphate-buffered saline (PBS, pH 7.2) and then resuspended (1.0×10^7^ cells/ml) in Czapek liquid broth. Suspensions containing 1.0×10^3^ cells/ml were added to the wells of a 24-well plate, followed by supplementation with aspartyl PIs at different concentrations: pepstatin A (1 and 10 µM), indinavir, saquinavir, ritonavir and nelfinavir (50, 100 and 200 µM). These mixtures were incubated for 1 hour at room temperature under constant agitation. Cells were then harvested by centrifugation, washed three times with PBS and re-inoculated in solid Czapek medium without drugs, to measure the CFU. All PIs were dissolved in absolute methanol. The solvent has also been tested for their possible ability to inhibit cellular growth, presenting negative results. A control was also made replacing PIs to citrate buffer [16]. In some experiments, the synergy susceptibility testing for aspartyl PIs and antifungal drugs (itraconazole or amphotericin B) were also evaluated, following the same above protocol. In this set of experiments, the conidia of *F. pedrosoi* were treated with both PIs (at 25 µM) and antifungal compounds (itraconazole at 0.3 µg/ml and amphotericin B at 3 µg/ml), alone or in combination, at sub-inhibitory concentration.

### Mammalian cells

CHO cells, provided by Dr. Pamela Stanley (Department of Cell Biology, Albert Einstein College of Medicine, NY, USA), RAW264.7 mouse macrophages was obtained from ATCC (code number TIB-71) and 3T3-L1 mouse fibroblasts were purchased from a Rio de Janeiro cell culture collection (BCR, registration number CR089). The mammalian cells were grown at 37°C with 5% CO_2_ in 25 cm^2^ culture flasks containing DMEM medium (Gibco), added with 10% fetal bovine serum (FBS) (Gibco). The pH was maintained at 7.2 by the addition of HEPES (3 g/l) and NaHCO_3_ (0.2 g/l) to the culture medium. The initial inoculum was 5.0×10^4^ cells/ml; cultures were subcultured every 2 days and the cells maintained in exponential growth phase.

### Fungus-host cells interaction

Animal cells were plated onto 24-well multidishes at a density of 1.0×10^5^ cells per well [24]. They were then incubated at 37°C for 24 hour in the presence of DMEM medium supplemented with 10% FBS. Before interaction with animal cells, conidia of *F. pedrosoi* (1.0×10^6^ cells) were incubated for 1 hour at room temperature in PBS (control) or in the same solution containing different concentrations (50, 100 and 200 µM) of HIV aspartyl-type PIs. Conidia were then washed twice with PBS and finally rinsed in DMEM. The fungal viability after these treatments presented more than 98% of survival. Fungal cells were suspended in the same medium to a ratio of 10 *F. pedrosoi* conidia per animal cell on monolayers. After the addition of *F. pedrosoi*, the cells were incubated at 37°C for 1 hour (macrophages) [25], [26] and 2 hours (fibroblast and CHO cells) [24], washed three times in PBS to remove non-adherent conidia, fixed in Bouin's solution and stained with Giemsa. The percentage of infected cells was determined by randomly counting a minimum of 300 cells on each triplicate coverslip and experiments were repeated at least 3 times. The adhesion index was calculated by multiplying the mean number of attached fungi per animal cell by the percentage of infected cells, observed by microscopic examination using an immersion objective in a Zeiss Axioplan 2 microscopy (Germany). Endocytic index was determined by the same way, but using the mean number of ingested fungi [24]. By means of optical and transmission electron microscopy (see below), representative images of the adhesion and endocytic processes were shown.

### Transmission electron microscopy

After the interaction of *F. pedrosoi* conidia-mammalian cells, the cultures were fixed for 1 hour, at room temperature, with a solution containing 2.5% glutaraldehyde, 4% paraformaldehyde, 10 mM CaCl_2_ in 0.1 M cacodylate buffer, pH 7.2, for 1 hour at 25°C. The cells were gently scraped off with a rubber policeman, washed with the same buffer and post-fixed for 1 hour in a solution containing 1% OsO_4_ and 0.8% potassium ferricyanide in cacodylate buffer. The cells were then rinsed, dehydrated in a graded series of acetone and embedded in Spurr resin. Ultrathin sections were obtained, stained with uranyl acetate and lead citrate, and examined in a JEOL 1200 EX transmission electron microscope [24]. Alternatively, *F. pedrosoi* conidia (1.0×10^3^ cells) pre-treated or not with the HIV aspartyl PIs at 100 µM for 1 hour were also processed to transmission electron microscopy, with the purpose of evidencing some ultrastructural alteration.

### Killing F. pedrosoi conidia by macrophages

First, the effect of the HIV aspartyl PIs on the macrophage (RAW264.7 lineage) viability was tested. In this sense, macrophages (1.0×10^5^ cells) were incubated in a 96-well cell culture plate for 24 hours in the absence or in the presence of aspartyl PIs at different concentration varying from 3.125 to 50 µM. Subsequently, the viability of macrophage cells was measured by MTT reduction assay. Briefly, each well was incubated with 0.5 mg/ml MTT for 3 hours at 37°C. Finally, cells were lysed by adding 0.6% acidic acid and 10% SDS in DMSO, to release and dissolve the formed formazan salt. The amount of incorporated formazan was determined spectrophotometrically at 490 nm. Second, in order to investigate the killing properties of RAW cells against conidia of *F. pedrosoi*, viable fungal cells were washed in PBS and allowed to interact with macrophages at a fungi-to-host cell ratio of 10∶1 during 1 hour at 37°C [25]. Nonassociated fungi were then removed by exhaustive washing in PBS and mammalian cells were incubated in DMEM medium for additional 24 hours in the absence or in the presence of HIV aspartyl PIs at 6.25 µM. Subsequently, the adhered macrophages were washed in PBS, lysed with sterile cold water and the resulting suspension was plated onto Czapek plates. After 7 days, the number of CFU was determined.

### Statistical analysis

All experiments were repeated at least three times. All systems were performed in triplicate, and representative images of these experiments are shown. The data were analyzed statistically using Student's *t* test using EPI-INFO 6.04 (Database and Statistics Program for Public Health) computer software. *P* values of 0.05 or less were considered statistically significant.

## Results

### Secretion of aspartyl peptidase from *F. pedrosoi* conidia

**Figure 1:**
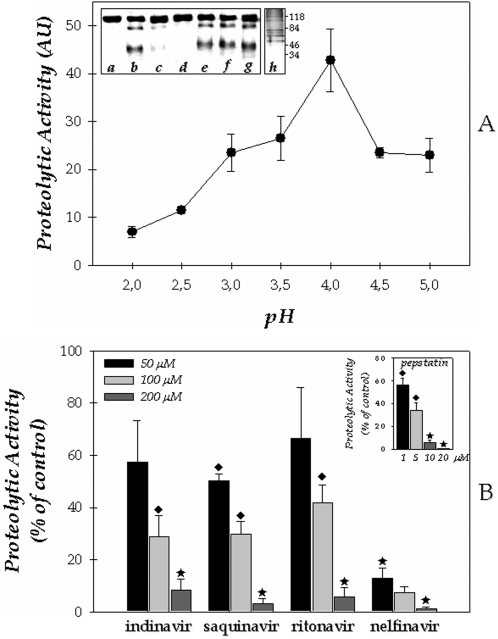
Biochemical properties of the secreted peptidase from conidial cells of *Fonsecaea pedrosoi*. (A) Effect of pH on the extracellularly released proteolytic activity of *F. pedrosoi* conidia. Cells were grown in Czapek chemically defined medium for 5 days at room temperature. Afterward, culture supernatant was filtered, concentrated and tested to degrade soluble BSA using 10 mM sodium citrate buffer adjusted for distinct pH's varying from 2.0 to 5.0. The values represent the mean±standard deviation of three independent experiments, which were performed in triplicate. The inset shows the effect of different PIs on the cleavage of BSA by *F. pedrosoi* conidial-derived peptidase. Concentrated supernatant was incubated for 1 hour at 37°C in 10 mM sodium citrate buffer, pH 4.0, in the absence (b) or in the presence of 1 µM pepstatin A (c), 10 µM pepstatin A (d), 1 mM PMSF (e), 1 mM E-64 (f) and 1 mM 1,10-phenanthroline (g). A control (a) in which the BSA was supplemented only with citrate buffer was used. These mixture reactions were applied on SDS-PAGE to detect the BSA hydrolysis, and the gel was stained with Coomassie blue R-250. In addition, the extracellular polypeptide profile secreted by *F. pedrosoi* conidia was analyzed by SDS–PAGE (h). In this last case, the gel was silver-stained and the numbers on the right indicate the relative molecular mass markers expressed in kilodaltons. (B) Effect of aspartyl PIs on the secretory peptidase activity from conidial forms of *F. pedrosoi*. Concentrated supernatant was incubated for 1 hour at 37°C in 10 mM sodium citrate buffer, pH 4.0, in the absence or in the presence of different concentrations of the following PIs: indinavir, saquinavir, ritonavir, nelfinavir (50, 100 and 200 µM) and pepstatin A (1, 5, 10 and 20 µM). The control (39.3±1.7 arbitrary units) was taken as 100%. The values represent the mean±standard deviation of three independent experiments performed in triplicate. Symbols denote systems treated with PIs that had a rate of substrate hydrolysis significantly different from the control (♦, *P*<0.05; ★, *P*<0.001; Student's t test).

Initially, we have investigated the secretion of proteolytic activity from *F. pedrosoi* conidial cells by testing the capability of the cell-free concentrated culture supernatant, obtained after growth in a chemically defined medium, to hydrolyze soluble albumin. Peptidase activity was detected under acidic conditions, presenting highest hydrolysis level at pH 4.0 (Fig. 1A). Subsequently, the effect of a number of classical PIs on the *F. pedrosoi* peptidase activity was tested. *F. pedrosoi* acidic extracellular peptidase activity was not inhibited by metallo (1,10-phenanthroline), serine (PMSF), or cysteine (E-64) PIs (Fig. 1A, inset). Conversely, the universal aspartyl PI pepstatin A at 10 µM totally inhibited the BSA cleavage (Fig. 1A, inset). Furthermore, we believe that the extracellular aspartyl peptidase activity is released and/or secreted by the conidial cells based on the fact that, within 5 days of *F. pedrosoi* cultivation in Czapek medium, more than 99% of conidial cells were viable when assessed by propidium iodide staining (data not shown). An inspection through SDS-PAGE revealed the polypeptide profile secreted by conidial cells into the culture supernatant medium, which was composed of seven major polypeptides with molecular masses ranging from 50 to 120 kDa (Fig. 1A, inset).

### Effect of HIV PIs on the extracellular proteolytic activity

The effect of distinct HIV aspartyl-type PIs on the extracellular proteolytic activity of *F. pedrosoi* conidial cells was also evaluated. To this end, saquinavir, ritonavir, nelfinavir and indinavir were tested at concentrations in the range of 50 to 200 µM. All four inhibitors restrained the conidial aspartyl proteolytic activity in a dose-dependent manner (Fig. 1B). Nelfinavir was by far the most effective inhibitor, reducing the secreted proteolytic activity by approximately 85% already at the lowest concentration used. Four-fold higher concentration of saquinavir, indinavir and ritonavir were necessary to obtain a similar inhibition pattern. In contrast, only a slight inhibition was detected when these three preceding inhibitors were tested at concentration of 50 µM (Fig. 1B). Overall, the inhibitory activities of the four HIV PIs at 200 µM were comparable to those exerted by pepstatin A (10 µM), a prototypal inhibitor of aspartyl-type peptidases (Fig. 1B, inset).

### Effect of HIV PIs on the growth

**Figure 2:**
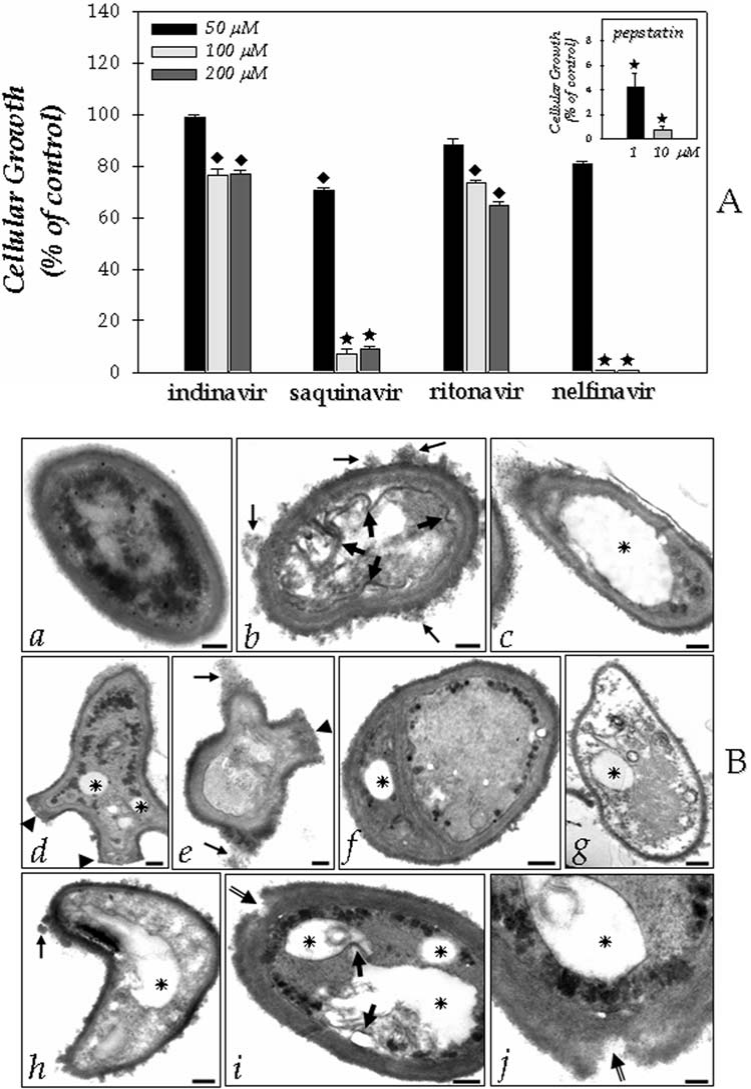
Functional implications of HIV PIs on the *Fonsecaea pedrosoi* conidial cells development. (A) Effect of aspartyl PIs on the *F. pedrosoi* conidial growth. After growth in Czapek medium for 5 days at room temperature, the conidia were harvested, washed with PBS and 1×10^3^ cells were incubated for 1 hour in the absence or in the presence of different concentrations of the following PIs: indinavir, saquinavir, ritonavir, nelfinavir (50, 100 and 200 µM) and pepstatin A (1 and 10 µM). After that, the conidial cells were washed in PBS and inoculated in a fresh solid Czapek medium to measure the CFU. The control (435.2±12.2 CFU) was taken as 100%. The values represent the mean±standard deviation of three independent experiments performed in triplicate. Symbols denote systems treated with PIs that had a growth rate significantly different from the control (♦, *P*<0.05; ★, *P*<0.001; Student's t test). (B) Effect of aspartyl PIs on the *F. pedrosoi* ultrastructure. After growth in Czapek medium for 5 days at room temperature, the conidia were harvested, washed with PBS and 1×10^3^ cells were incubated for 1 hour in the absence (a) or in the presence of nelfinavir (b–g) or saquinavir (h–j) at 100 µM. Subsequently, the cells were processed by transmission electron microscopy as described in the Experimental Procedures section. Control conidial cells present a characteristic dense cytoplasm with a distinct and compact cell wall (a). Large arrows in (b) and (i) show the invaginations of cytoplasmic membrane. Tiny arrows in (b), (e) and (h) indicate the disorder and detachment of cell wall. Arrowheads in (d) and (e) demonstrate the abnormal cellular division. Asterisks show the enlarged cytoplasmic vacuoles. Double arrows in (i) and (j) appoint the breakage of cell wall starting from extracellular medium. Note in (g) a typical necrotic conidial cell. Scale bars: (a–i), 0.6 µm; (j), 0.15 µm.

In order to establish whether the aspartyl PIs might have any effect on *F. pedrosoi* development, we performed experiments in which conidia (1.0×10^3^ cells) were assayed for growth ability, by CFU assay, following 1 hour exposure to HIV PIs (50, 100 and 200 µM) or pepstatin A (1 and 10 µM). Concentrations of 50 µM of each PIs did not significantly reduce the conidial development (*P*>0.05), except for saquinavir (*P*<0.05). Conversely, nelfinavir at 100 µM completely abolished cellular growth, indicating a complete cure of the *F. pedrosoi* conidia culture by this HIV PI. Saquinavir also drastically reduced the cellular growth at 100 µM by approximately 90% (Fig. 2A). Similarly, pepstatin A powerfully diminished the conidial growth even at 1 µM (Fig. 2A, inset). Conversely, indinavir and ritonavir at the highest concentration used only diminished the growth of *F. pedrosoi* in the order of 20–30% (Fig. 2A). At a concentration of HIV PIs equal or lower than 25 µM, no effect on the growth of *F. pedrosoi* conidial cells was observed (data not shown).

### Effect of HIV PIs on the ultrastructure

Based on the efficacy of the HIV aspartyl PIs in diminishing the growth of *F. pedrosoi*, particularly nelfinavir and saquinavir, we next investigated the effect of both inhibitors on the *F. pedrosoi* morphological level (Fig. 2B). In this context, by means of transmission electron microscopy, the normal morphology of control conidium (Fig. 2B, panel a) was compared with the ultrastructure of PIs-treated cells (Fig. 2B, panels b–j). After 1 hour of treatment, both nelfinavir and saquinavir at 100 µM induced several morphological alterations, including: invaginations in the cytoplasmic membrane and withdrawal of the cytoplasmic membrane from within the cell wall (large arrows in Fig. 2B, insets b and i), disorder and detachment of the cell wall (tiny arrows in Fig. 2B, panels b, e, and h), rupture of internal organelles (Fig. 2B, panel g), and the presence of large and irregular cytoplasmic vacuoles (asterisks in Fig. 2B), some of them containing small vesicles (Fig. 2B, panels i and j). In some cells, the enlargement of intracellular vacuoles was so intense that they occupied almost the entire cytoplasm area (Fig. 2B, panel c). Furthermore, abnormal division disfiguring regular conidia morphology could be observed in cells treated with nelfinavir (arrowheads in Fig. 2B, panels d–f), while the saquinavir treatment seams to induce breakage of cell wall starting from extracellular environmental (double arrows in Fig. 2B, panels i and j). Ritonavir and indinavir also promoted some of these aberrant alterations; however, in a lower proportion when compared to nelfinavir and saquinavir treatment (data not shown). In contrast, non-treated conidial cells presented a normal morphology with a characteristic dense and dark cytoplasm and a distinct cell wall (Fig. 2B, panel a).

### Effect of HIV PIs on the interaction of conidia-animal cells

**Figure 3:**
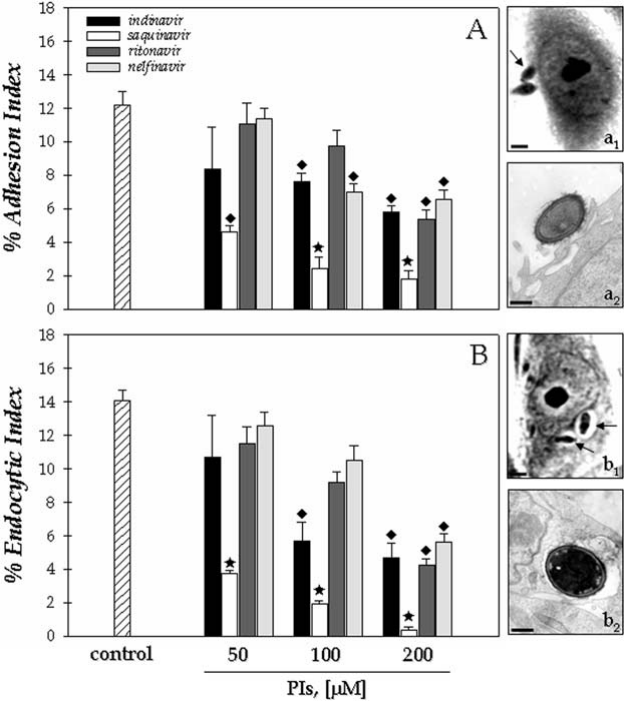
Effect of aspartyl HIV PIs on the interaction between *Fonsecaea pedrosoi* conidia and epithelial cells (CHO). After growth in Czapek medium for 5 days at room temperature, the conidia were harvested, washed with PBS and 1×10^6^ cells were incubated for 1 hour in the absence or in the presence of different concentrations (50, 100 and 200 µM) of the following PIs: indinavir, saquinavir, ritonavir and nelfinavir. After that, the conidial cells were washed in PBS and the interaction process was performed as described in Experimental Procedures section. The viability of the conidial cells was not affected by the PIs treatment used in this set of experiments as judged by propidium iodide staining (data not shown). Adhesion (A) and endocytic (B) indices of the interaction after 2 h were shown. The values represent the mean±standard deviation of three independent experiments performed in triplicate. Symbols denote systems treated with PIs that had an interaction index significantly different from the control (♦, *P*<0.05; ★, *P*<0.001; Student's t test). Representative images of attached (a_1_ and a_2_) and ingested (b_1_ and b_2_) fungi are shown by means of light (a_1_ and b_1_) and transmission electron (a_2_ and b_2_) microscopy analyses. Note that ingested conidia are located within vacuole (b_1_ and b_2_). Arrows show the conidia attached (a_1_) or ingested (b_1_) by CHO cells. Scale bars: (a_1_ and b_1_), 10 µm; (a_2_ and b_2_), 1 µm.

Light microscopy of Giemsa-stained preparations, revealed that conidia of *F. pedrosoi* attached to (Fig. 3A, a_1_), and were ingested (Fig. 3B, b_1_) by Chinese hamster ovary cells (CHO) after 2 hours of fungus-mammalian cell contact as previously experimented [24]. Transmission electron microscopy analysis showed that the fungus adhered to cell surface by a discreet contact between its cell wall and the cytoplasmic membrane of the vertebrate cell (Fig. 3A, a_2_). After ingestion, conidial cell could be clearly observed within a membrane cytoplasmic vacuole by using both light and electron microscopy (Fig. 3B, b_1_–b_2_). Then, we evaluated the influence of HIV PIs on this interaction process. In this sense, conidia (1.0×10^6^ cells) were pre-treated with each aspartyl PI (50, 100 and 200 µM) for 1 hour before interaction. In this cellular density, the conidia maintained their viability after the treatment for 1 hour with each PI in all the studied concentration as judged by their morphology and propidium iodine staining (in which more than 99% of the conidia were viable). Similar results were obtained by our group when different cell numbers were treated with pepstatin A [16]. In the present study, we showed that saquinavir was the most potent inhibitor of both adhesion and endocytosis processes between *F. pedrosoi* conidia and CHO cells, showing a clear dose-dependent inhibition profile, where the inhibition increased from 60 to 85% (adhesion index) and 70 to 97% (endocytic index) as saquinavir concentration rose from 50 to 200 µM (Fig. 3). Curiously, nelfinavir, which most effectively inhibited both secreted aspartyl peptidase and cellular growth, only significantly reduced the adherence and endocytosis of *F. pedrosoi* to 55% of the controls at highest concentration. Ritonavir and indinavir both at 200 µM diminished the interaction process in a similar way (Fig. 3).

**Figure 4:**
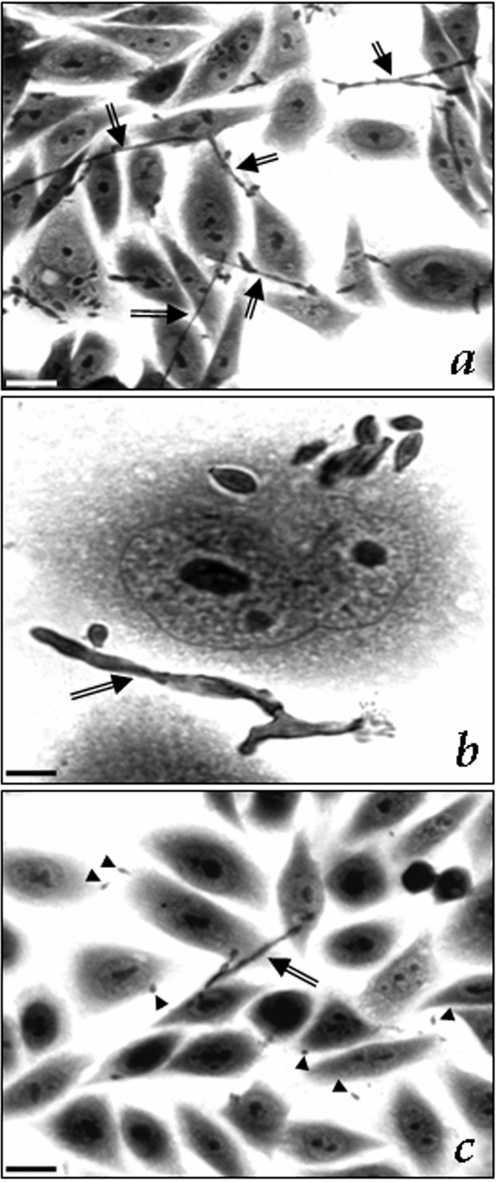
Effect of aspartyl HIV PIs on the *F. pedrosoi* morphological transition (conidia into mycelia transformation) after the interaction with epithelial cells (CHO). In this set of experiment, conidia of *F. pedrosoi* were initially untreated (a and b) or treated (c) with 100 µM of HIV aspartyl PIs for 1 hour, washed in PBS and then interacted with CHO cells for 4 hours. Optical microscopy of the interaction showed that in the control system, some conidial cells transform in hyphae, as indicated by the arrows, showing the well-known differentiation process triggered by the contact of conidia with host cells. Conversely, when conidia were pre-treated with aspartyl PIs, especially saquinavir (c), the number of hyphae is drastically diminished. Arrowheads show the conidia. Scale bars: (a, c), 10 µm; (b), 1 µm.

Interestingly, if the interaction process was examined after 4 hours of contact fungi-CHO cells, we observed a great amount of mycelium, showing the typical conidia differentiation when in contact with animal cells (arrows in Fig. 4). On the other hand, the pre-treatment of conidia for 1 hour with the HIV aspartyl PIs, principally saquinavir, drastically blocked the conidia into mycelia transformation (compare Fig. 4, panels a and c).

**Figure 5:**
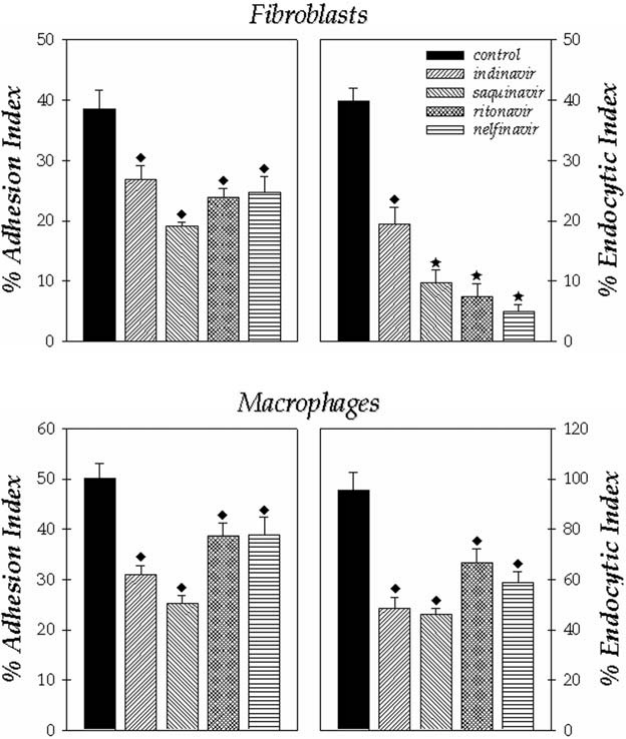
Effect of aspartyl HIV PIs on the interaction between *F. pedrosoi* conidial cells and fibroblasts or macrophages. After growth in Czapek medium for 5 days at room temperature, the conidia were harvested, washed with PBS and 1×10^6^ cells were incubated for 1 hour in the absence or in the presence of the following PIs at 200 µM: indinavir, saquinavir, ritonavir and nelfinavir. After that, the conidial cells were washed in PBS and the interaction process was performed as described in Experimental Procedures section. The viability of the conidial cells was not affected by the PIs treatment used in this set of experiments as judged by propidium iodide staining (data not shown). Adhesion and endocytic indices of the interaction with macrophages for 1 hour or fibroblasts for 2 hours were shown. The values represent the mean±standard deviation of three independent experiments performed in triplicate. Symbols denote systems treated with PIs that had an interaction index significantly different from the control (♦, *P*<0.05; ★, *P*<0.001; Student's t test).

We also evaluated the effect of these PIs on the *F. pedrosoi* conidia during the interaction with two other cell lineages, fibroblasts and macrophages. In relation to fibroblasts, the endocytic index was more affected by the treatment of conidial cells with the inhibitors than the adhesion one, in which nelfinavir, saquinavir and indinavir in the same way diminished the process by approximately 80% (Fig. 5). In respect to macrophages, the treatment of conidial cells with indinavir and saquinavir produced the best inhibition profiles in comparison with control, promoting a declining on both adhesion and endocytic indices by around 50% (Fig. 5).

It is important to elucidate that the earlier steps of endocytosis, such as adherence of the fungus on the surface of mammalian cells, pseudopodia formation (in the case of macrophage cells), and internalization of fungus seemed to be similar in both untreated and aspartyl PI-treated conidial cells (data not shown).

### HIV PIs improve the macrophage killing against *F. pedrosoi*

**Figure 6:**
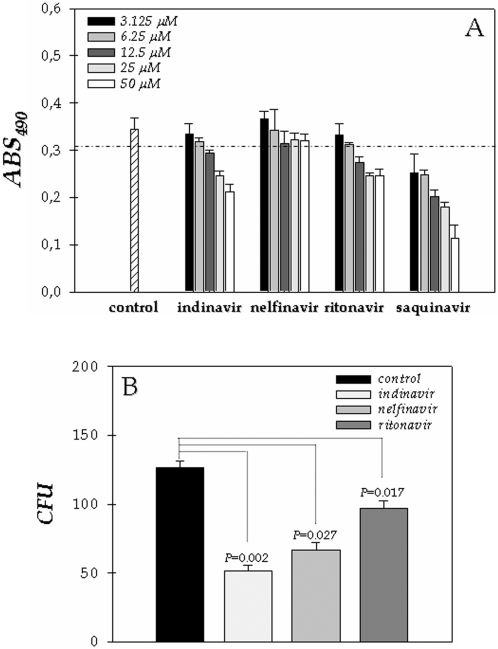
Effect of aspartyl PIs on the viability and killing activity of macrophage cells (RAW264.7 lineage). Initially, the macrophages (1×10^5^ cells) were incubated in a 96-well plate for 24 hours in the absence or in the presence of different concentrations (3.125, 6.25, 12.5, 25 and 50 µM) of the following PIs: indinavir, saquinavir, ritonavir and nelfinavir. After this period, the viability of the macrophage cells was determined spectrophotometrically at 490 nm by means of MTT assay. The dotted line separates the graphic in two portions: minor than and equal or major than 90% of macrophage viability (A). After that, we studied the influence of aspartyl PIs on the killing activity of macrophages. In this context, we repeated the interaction of conidia with macrophage (ratio of 10∶1, respectively) for 1 hour, followed by exhaustive washing in PBS and then incubated the interaction for additional 24 hours in the absence (control) or in the presence of indinavir, nelfinavir and ritonavir at 6.25 µM, concentration in which more than 90% of the macrophage cells were viable as demonstrated in (A). Finally, the adhered macrophages were washed, lysed with sterile cold water, and the resulting suspension was plated on Czapek solid medium to count the number of CFU (B).

*F. pedrosoi* is able to completely destroy the host macrophage within 24 hours of interaction, when macrophage debris and membrane ruptures can be observed [25], [26]. On the other hand, the improvement in macrophage function during HAART of HIV-infected patients was previously documented [19]. With these informations in mind, we resolved to investigate the killing capability of macrophage against *F. pedrosoi* during the in vitro treatment for 24 hours with aspartyl PIs used in the chemotherapy of HIV. First of all, we tested the effect of these four PIs alone on the macrophage (RAW264.7 lineage) viability by using the MTT assay. Our results showed that all the PIs diminished the macrophage viability after 24 hours of contact, in a dose-dependent fashion (Fig. 6A). Therefore, in the next section of these experiments, we used the inhibitors at 6.25 µM, concentration in which more than 90% of macrophages were viable after 24 hours of treatment (Fig. 6A). Saquinavir was not applied in killing assay, since even at the lowest concentration (3.125 µM) this inhibitor reduced considerably the viability of macrophage cells (Fig. 6A), dropping the number of viable intracellular conidia.

In order to verify whether macrophage killing is altered by HIV aspartyl-type PIs, macrophages were infected for 1 hour with *F. pedrosoi* conidia (1 macrophage: 10 conidia) and then the infected cultures were exhaustively washed and incubated for 24 hours in the absence or in the presence of indinavir, nelfinavir and ritonavir at 6.25 µM. After this period, macrophages were washed, lysed and the number of viable fungi determined by CFU counts. Our results evidenced that the aspartyl PIs notably enhanced the antifungal action of macrophages, principally indinavir that reduced the intracellular conidia viability around 60% (Fig. 6B).

### Synergist effect of HIV PI and antifungal drugs on the *F. pedrosoi* growth

**Figure 7:**
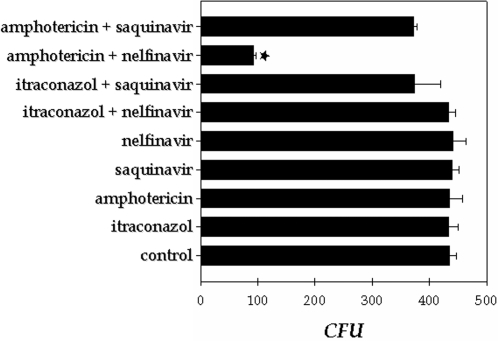
Study of the possible synergistic effect between aspartyl HIV PI and antifungal compounds against *F. pedrosoi* development. In this experiments, conidia (1×10^3^ cells) were treated or not with a sub-inhibitory concentration of both PIs (saquinavir and nelfinavir at 25 µM) and antifungal drugs (itraconazol at 0.3 µg/ml and amphotericin B at 3 µg/ml), alone or in combination for 1 hour. Afterward, the conidial cells were washed in PBS and inoculated in a fresh solid Czapek medium to measure the CFU. The values represent the mean±standard deviation of three independent experiments performed in triplicate. Symbol denotes system that had a growth rate significantly different from the control (★, *P*<0.001; Student's t test).

Unfortunately, the PIs can generate adverse effects and these, with drug interactions, may restrict their clinical use [27]. So, the possible use of aspartyl PIs at concentrations below the range of effective antimicrobial activity in combination with the standard antifungal drugs to kill conidial cells was also evaluated. In this set of experiments two antifungal agents (itraconazole and amphotericin B) and the two most potent aspartyl PIs of conidial growth (saquinavir and nelfinavir) were tested. As expected, when used at sub-inhibitory concentration, both antifungal compounds (itraconazole at 0.3 µg/ml and amphotericin B at 3 µg/ml) and PIs at 25 µM alone did not diminish the conidial growth after 1 hour of treatment (Fig. 7). However, when amphotericin B and nelfinavir were combined, fungal killing was close to 72% (Fig. 7). This last result indicated that both antimicrobial compounds act synergistically to kill *F. pedrosoi* conidial cells.

### Secretion of aspartyl peptidase by different clinical isolates of *F. pedrosoi*

In order to demonstrate that the secretion of aspartyl peptidase activity is a general feature by conidial cells of *F. pedrosoi* species instead of being strain-specific, we analyzed the extracellular peptidase content in two other distinct clinical isolates of *F. pedrosoi* (11428 and Magé strains). Herein, the cells were grown in chemically defined conditions for 5 days and the conidial-free concentrated supernatants were used to measure the proteolytic activity. The characteristics of the isolates and comparative quantification of extracellular proteolytic activities are summarized in Table 1. Interestingly, the *F. pedrosoi* Magé strain, which was recently isolated by our group, produced approximately 1.3- and 2-fold higher levels of extracellular proteolytic activity when compared with the 5VPL and 11428 strains, respectively (Table 1), suggesting that the production of secreted aspartyl-type peptidases may be stimulated by interaction with the host.

**Table 1:** Extracellular proteolytic activity in different clinical isolates of *Fonsecaea pedrosoi*.

Strain	Time of isolation	Number of in vitro passages	Proteolytic activity (AU) a	
			control	+pepstatin A (1 µM)
5VPL	30 years	>500	39.3±1.7	0.8±0.5
11428	2 years	8	26.4±4.8	4.8±2.8
Magé	6 months	6	51.8±5.3	5.3±1.8

a The proteolytic activity was quantified in the absence (control) or in the presence of pepstatin A in the standard assay described in Experimental Procedures section using BSA as soluble proteinaceous substrate, 10 mM citrate buffer, pH 4.0, and 20 µl of each concentrated supernatant (equivalent to 5 µg of protein). The proteolytic activity was expressed as arbitrary unit (AU).

## Discussion

The evolution of fungal infections has significant clinical repercussions and the prevalence of fungal diseases has increased markedly in hospitalized patients and other individuals with immune impairment, including those receiving solid-organ or bone-marrow transplants, those with hematological malignancies, patients on long-term corticosteroids and patients with uncontrolled or undiagnosed HIV infection [28], [29]. Patients with HIV infection are surviving longer and with an improved quality of life as a result of the institution of HAART, regimens for which usually consist of a PI combined with at least two other antiretroviral drugs [30], [31]. PIs bind competitively to the cleavage site within the HIV-1 aspartyl peptidase enzyme. They render the enzyme non-functional and lead to the release of immature, non-infectious viral particles [32]. Moreover, it has been demonstrated that PIs have advantageous effects on some opportunistic infections caused by fungal pathogens, known to be the major causes of morbidity and mortality in AIDS subjects, by both restore the host immune system and to direct action on the pathogen. Based on the preceding evidences, we studied the implication of four distinct HIV PIs on the human fungal pathogen *F. pedrosoi*, focusing on the effects in the extracellular peptidase activity, development and interaction with animal cells.

Extracellular peptidases of eukaryotic microbial pathogens have attracted the attention of many laboratories because of their potential role in pathogenesis. They facilitate the penetration into the host organism and counteract its defense system. Analysis of proteolytic enzymes of pathogenic microorganisms might lead to a design of inhibitors controlling these pathogens. As previously reported by our group, *F. pedrosoi* conidial cells produce and release aspartyl-type peptidase into the extracellular environment when cultured under chemically defined conditions able to degrade several key host proteins [16], [17]. The aspartic peptidases are a family of enzymes involved in a number of important biological processes [33]. Consequently, aspartic peptidases are already the targets of some clinically useful drugs and a variety of factors make these enzymes appealing to those seeking novel antifungal therapies. In the present work, we confirmed that *F. pedrosoi* are able to secrete aspartyl peptidase and additionally we showed that the hydrolytic activity was best active at pH 4.0 and sensitive to pepstatin A, in a dose-dependent fashion.

Pepstatin-like drugs, however, are not used clinically because of their metabolism in the liver and rapid clearance from blood [34]. This led to recent studies showing that HIV PIs have indeed a direct attenuating effect on *Candida albicans* Saps in vitro and in vivo [12], [35], [36], [37] as well as on *Cryptococcus neoformans* [38], [39]. So, we tested the effect of HIV PIs on the aspartyl peptidase extracellularly released by *F. pedrosoi* conidia cells in vitro. Nelfinavir seems to be the most active in inhibiting *F. pedrosoi* secreted aspartyl peptidase, whereas saquinavir, indinavir and ritonavir also restrained the peptidase activity in a lower fashion. Thus, the present data imply a direct inhibitory effect of HIV PIs on the *F. pedrosoi* conidial extracellular aspartyl-type peptidase.

*F. pedrosoi* was also exposed to pepstatin A, nelfinavir, indinavir, saquinavir and ritonavir for 1 hour, and then the fungal survival was analyzed. As previously reported by our group, pepstatin A promoted a powerful reduction on the growth of *F. pedrosoi* conidia, but this fungicidal action was dependent on the cell number, since 10^6^−10^4^ conidia were not affected by 10 µM of pepstatin while 10^2^−10^3^ conidial cells were fully inhibited [16]. Concerning cell density, similar results were also observed with the HIV aspartyl PIs (data not shown). Nelfinavir and saquinavir robustly inhibited the *F. pedrosoi* growth at 100 µM, in contrast to ritonavir and indinavir. It is also important to note that the inhibitory effect of HIV PIs in vitro and in candidal vaginitis were observed at substantial PI concentrations (50–200 µM), much higher than those needed for HIV peptidase inhibition (of the nM order) [40]. This probably reflects a much lower affinity of these drugs for Sap compared with their high affinity for HIV-1 aspartyl peptidase [40]. Although the above interpretation of in vitro anti-*F. pedrosoi* effects of HIV PIs appears to be coherent with all experimental data, the possibility of unrelated secreted aspartyl peptidase effects of inhibitors on the *F. pedrosoi* should be considered, such as nonspecific or generally toxic effects on conidial cells. In this context, electron-micrographic examination of fungal cells exposed to saquinavir and nelfinavir revealed some peculiar alterations in vital cellular structures of *F. pedrosoi* conidia, such as cell wall, cytoplasmic membrane and internal cellular structures, suggesting irreversible metabolic injury that culminates in the conidial cell death.

The process of penetration of microorganism into host cells begins with adhesion to the host cell surface and is followed by its internalization. Adhesion of *F. pedrosoi* is known to be a complex, multifactorial property dependent on a multiplicity of recognition systems and surface receptors such as a lectin-like molecule [41] and an ecto-phosphatase [42]. Additionally, general biophysical properties of the cell such as cell surface hydrophobicity and electrostatic charge can affect adhesion [reviewed in 43]. Peptidases can also modulate the binding of fungal cells to surface of the host, suggesting a possible non-enzymatic role for this class of hydrolytic enzymes in adherence [15]. Our results showed that after the treatment of *F. pedrosoi* cells for 1 hour with the PIs a noticeably dose-dependent inhibition was observed on both adhesion and endocytic indexes when the fungus was interacted with epithelial, fibroblast and macrophage cell lineages. These interaction inhibition profiles were not caused by diminishing the viability of *F. pedrosoi* cells, as demonstrated by propidium iodide staining. However, by what molecular mechanism the secretory peptidase mediates adhesion remains on open question. In analogy to the results described early for *Candida albicans*, two hypotheses are possible: the extracellular peptidase could act as ligand to surface proteins of the specific host cells, which does not necessarily require their enzymatic activity, and the fungal cells use secreted peptidase as active enzymes to affect target structures of their host cells, leading to a conformational change of the surface proteins or ligands of host cells allowing a better adherence of the fungal cells [reviewed in 15].

A crucial event during *F. pedrosoi* life cycle is its differentiation process, in which any of the fungal forms (conidia, mycelia and sclerotic cells) can generate all of the others, except for the transition of sclerotic cells into conidia [44]. As early proposed, the understanding of this process and, consequently, how this morphological transition can be inhibited may allow the design of alternative and efficient therapies against chromoblastomycosis [3]. As well, it is widely recognized that formation of hyphae enhances fungal adherence and tissue invasion, with more efficient release of proteolytic activity [18]. Interestingly, when *F. pedrosoi* conidia were pre-treated for 1 hour with the aspartyl PIs and then interacted for 4 hours with epithelial cells in vitro, we observed a considerably inhibition of the conidia into mycelia transformation. Probably the aspartyl peptidase may be involved in the early transformation or remodeling of the conidia after it enters the host cells. Conidia to mycelia transformation is accompanied by dramatic morphologic changes that could require peptidase-mediated degradation of several proteins, including conidia cytoskeleton components.

Previous studies demonstrated that *F. pedrosoi* is able to survive and proliferate in tissue macrophages and that activated macrophages are fungistatic but not fungicidal [25], [26]. The virulence of this microorganism is directly correlated to its ability to survive and multiply intracellularly. In the present work, we demonstrated that the presence of PIs during the infection of macrophages by *F. pedrosoi* conidia for 24 hours enhanced the antifungal properties of phagocytic cells, promoting a significantly reduction in the number of viable intracellular conidia. Based on these evidences, we can speculate that besides their direct antifungal action, the PIs could block the conidia into mycelia transformation and consequently impairing the lysis of macrophages. Curiously, the direct effect of indinavir on the *F. pedrosoi* growth was modest with respect to other PIs; conversely, indinavir produced a very remarkable antifungal activity of macrophage cells. The augmented susceptibility of indinavir-treated *F. pedrosoi* to intracellular killing by macrophages could be correlated with improving production of oxygen reactive species by these cells. As is well-known, the respiratory burst activity represents one of the most important mechanisms mediating the antimicrobial immunity of phagocytic cells [9]. Furthermore, as previously reported by our group [16], [17], secreted aspartyl-type peptidases from *F. pedrosoi* conidial and mycelial cells have been considered as potential virulence factors capable of interfering with host defense mechanisms by affecting the integrity of relevant host proteins, and indinavir is able to inhibit these hydrolytic activities annulling their possible destructive effects. As a result, the constant presence of the PIs enhances the mammalian macrophage killing capability, suggesting their potential as a useful tool to control experimental fungal infections. Nevertheless, it remains unknown whether these PIs are active in vivo, since such studies are impaired by the lack of a reproducible animal model of chromoblastomycosis. Other researcher groups described related results. For instance, indinavir selectively inhibited production of some virulence factors of *Cryptococcus neoformans*, such as urease and extracellular peptidase [39]. Additionally, indinavir also interfered with polysaccharide capsule formation, the major virulence factor produced by this opportunistic fungal pathogen. Collectively, these deleterious effects led to increased susceptibility of *Cryptococcus neoformans* to intracellular killing by natural effector's cells, including microglial cells, blood mononuclear cells and polymorphonuclear leukocytes [38], [39].

Several treatment regimes have been used to treat chromoblastomycosis but long-term efficacy is still too low to allow specialists to elect a drug of choice. Itraconazole (200–400 mg per day for a long period, usually between 6 and 12 months) is considered as an alternative; however, many patients do not show clinical improvement [6]. For some cases, the best therapeutic strategy in cases of chromoblastomycosis seems to be a combination of two drugs chosen according to the results of prior antifungal susceptibility testing [45], such as the combination of amphotericin B and 5-fluorocytosine, itraconazole and 5-fluorocytosine [46], or itraconazole and terbinafin [47]. For this reason, we also studied the effects of the combination of antifungal and aspartyl PI drugs on the *F. pedrosoi* development. Under our experimental conditions, the results obtained from the combination at sub-inhibitory concentration of amphotericin B with sub-inhibitory concentration of nelfinavir indicated a good synergistic action in vitro, enhancing the antifungal activity of these drugs that occasioned a powerful inhibition of the conidia growth. Comparable results were obtained for the combinations of paromomycin and individual PIs (indinavir, ritonavir and saquinavir) on the inhibition of *Cryptosporidium parvum* growth in vitro [48] and fluconazole/saquinavir combined treatment for *Candida albincans* and *Cryptococcus neoformans* [49]. The advantage of the synergic effect of the combination is to attenuate the resistance and accompanying toxic phenomena, particularly and above all in the event of long-term therapy thank to the use of low dosages of both PIs and antimycotic drugs.

Taken together, previous and present data corroborate for the wide-spectrum activity of HIV aspartyl PIs, being them effective also on microorganisms phylogenetically distant from HIV, such as *F. pedrosoi* (present study), *Candida albicans* [reviewed in 15], *Cryptococcus neoformans* [38], *Cryptosporidium parvum* [48], *Pneumocystis carinii* [50] and *Leishmania* spp [51]. In conclusion, we provide evidence that the HIV PIs have multiple effects on crucial biological processes of *F. pedrosoi*, including cellular growth, differentiation and interaction with mammalian cell lineages. HIV PIs are also able to block the extracellularly released aspartyl-type peptidase produced by *F. pedrosoi*, which is a wide spectrum hydrolytic enzyme capable in cleaving several protein components. Furthermore, an improvement in the antifungal killing response of macrophages was noticed, supporting the possibility of exploiting HIV PIs as a potential therapeutic agent, alone or in combination with currently antimycotic drugs, in chromoblastomycosis. Consequently, *F. pedrosoi*-derived aspartyl peptidase is a promising target for rational drug design. However, additional studies will be needed to identify *F. pedrosoi* aspartyl peptidase and to find new PIs that are more specific for this fungal hydrolytic enzyme, leading to their use as alternative and novel topical therapeutic agents.
